# Potential predictors of length of study for finance and accounting degrees: evidence from a public business school in Saudi Arabia

**DOI:** 10.1016/j.heliyon.2022.e09636

**Published:** 2022-06-03

**Authors:** Nassar S. Al-Nassar, Ahmed A. Alhajjaj, Abbas Bleady

**Affiliations:** a Department of Economics and Finance, College of Business and Economics, Qassim University, Buraydah, Saudi Arabia; b Department of Management Information Systems and Production Management, College of Business and Economics, Qassim University, Buraydah, Saudi Arabia

**Keywords:** Graduation, Timely graduation, Time to degree, Length of study, Duration of study, Saudi business college, Multinomial logistic regression, Odds of graduation

## Abstract

The accumulated evidence from developed countries indicates that a large proportion of undergraduates exceed the normal time to obtain their degrees before completing their baccalaureate studies, which has attracted the attention of academics and policy-makers. However, the evidence on degree completion in developing countries is scant to nonexistent. The present study aims to fill this gap by developing a predictive model to explore the impact of the student's preadmission criteria and academic performance indicators on the study length for graduates of the bachelor of business administration (BBA) degree in finance and accounting in a Saudi public university. We used deidentified demographic and academic data from the 2018/2019 cohort of students at the College of Business and Economics (CBE), Qassim University. The dataset is assembled from official administrative student records. Using multinomial logistic regression (MLR), we find that students with a higher college entry age, higher secondary school score percentage, higher General Achievement Test (GAT) score, and higher academic performance in “gatekeeper” quantitative courses, including mathematics, statistics and economics, are more likely to graduate within the normal time to degree. The implications of the findings and future research directions are discussed.

## 1 Introduction

Baccalaureate degree completion, particularly in a timely manner, is an important concern in higher education. The increased time-to-degree in the US over the past three decades has recently attracted public policy attention (Bound et al., 2012). The fact that graduation delays are more prevalent in public US universities than in their private counterparts has led to the implementation of new regulations that link higher education funding to improved graduation rates (Stohs and Schutte, 2019). Timely graduation is associated with a plethora of benefits to the greater society. From the perspective of administrators, timely graduation prevents the draining of resources by allowing institutions to accommodate new students so that enrollment can continue to grow (Zhu, 2004). Moreover, prestigious university ranking agencies assign a high weight to the ability of institutions to retain and graduate students in normal time.^1^

With regard to student perspectives, Tinto (1987) posits that students are constantly trading off resources meant for a college education (opportunity costs) against future economic benefits they might accrue from attaining a bachelor's degree. Thus, an earlier graduation can reduce opportunity costs while increasing the possibility of obtaining economic returns from education. Tertiary attainment is also linked with a variety of socioeconomic benefits, including more tax revenue, as college graduates earn double the salary of secondary school-educated workers and six times as much as secondary school dropouts (Murphy and Welch, 1993). In addition, the spouses of college graduates are more educated, and their offspring are more likely to excel at school and less likely to commit crimes (Jencks and Edin, 1995; Murphy and Welch, 1993).

While several studies have been conducted to understand the determinants of timely graduation in the US (Bound et al., 2012; Huntington-Klein and Gill, 2020; Kurlaender et al., 2014; Stohs and Schutte, 2019; Witteveen and Attewell, 2021; Yue and Fu, 2017; Zhu, 2004), fewer studies have been carried out in developed countries in Europe and the rest of the world, including Lassibille and Navarro Gómez (2011) in Spain and Aina et al. (2011) and Brugiavini et al. (2020) in Italy. To date, this issue has not received adequate attention in developing countries. To the best of our knowledge, no study has systematically explored time to degree and the factors that impact students' progress toward the completion of their program of study in developing countries.

Saudi Arabia stands out among other developing countries by achieving unparalleled development in its higher education system in a short period of time (Saleh, 1986; Smith and Abouammoh, 2013). In addition, Saudi Vision 2030 intends to create a globally competitive and adaptive human resource base to support its ambitious plans to transform its economy from an oil-based to a knowledge-based economy (Amirat and Zaidi, 2020; Nurunnabi, 2017). Therefore, the role of higher education institutions in effectively qualifying graduates in a timely manner is more important than ever before. According to the Organization for Economic Cooperation and Development (OECD (2019), tertiary attainment is lower in Saudi Arabia than in most OECD and partner countries; however, tertiary education rates are expected to increase. In 2017, the first-time entry rate into bachelor's programs was 66%, which was higher than the OECD average of 58%. However, the goal of a college education is not access to a program of study per se, but rather an earned degree.

Furthermore, the emergence of the new Law of Universities fosters the autonomy of Saudi universities and removes foreign university entry barriers to enhance competition and raise the efficiency of the higher education system. Under the new law, universities are required to maintain academic accreditation from a national accreditor or from an internationally renowned accreditation body. Moreover, all academic units are required to disclose key performance indicators (KPIs) and demonstrate continuous improvement (Ministry of Education (MOE), 2020). Therefore, making available reliable and detailed data about students’ academic progression toward their degree completion is no longer a choice for colleges and schools in Saudi Arabia.^2^ Those institutional features in Saudi Arabia provide unique settings to extend prior studies by providing further insights into the determinants of time to degree in public colleges.

We exploit this opportunity to contribute to the literature by building a predictive model to explore the determinants of time to degree for the BBA in Accounting and Finance programs’ graduates at the College of Business and Economics (CBE) at Qassim University (QU). The choice of business students is motivated by the fact that accepted students in business schools constitute the largest portion of enrollment compared to other schools within Saudi universities. Furthermore, business schools are accessible for both male and female students (Alsudairi, 2015; Sian et al., 2020), unlike schools that are traditionally male (female) dominated, such as engineering (nursing) schools (Alghamdi et al., 2018; Labib et al., 2021). Indeed, The respective business school (the CBE at QU) from which we draw our sample is one of the few Saudi business schools that earned the prestigious Association to Advance Collegiate Schools of Business (AACSB) business accreditation. Thereby, CBE is expected to design its degree programs, including the normal time to degree, to ensure attainment of high-quality learning outcomes in relation to the assurance of degree program structures and design standards as prescribed by the AACSB (The Association to Advance Collegiate Schools of Business (AACSB), 2018). The choice of accounting and finance majors stems from the increased popularity of accounting and finance majors among Saudi students. The interest of students in these majors is driven by the potential increase in demand in the job market for qualified national graduates in those specializations due to the Saudi government initiatives to develop the financial sector (Grand and Wolff, 2020) and the recent rules and regulations meant to increase the participation of Saudi professionals in the financial sector and accounting profession (Saudi Gazette, 2020).

On a methodological front, this study employs a quantitative design by making use of secondary data on students' preadmission criteria (including demographics and quantitative preadmission criteria) and students' academic performance indicators to examine the impact of these variables on time to degree. The entire dataset is collected from official administrative students’ records of the CBE at QU rather than using a survey, in the spirit of recent studies examining the determinants of time to degree (Demeter et al., 2022; Fischer et al., 2021).

The main findings that emerge from our analysis are that students with a higher college entry age, higher secondary school score percentage, higher General Achievement Test (GAT) score, and higher academic performance in “gatekeeper” quantitative courses including mathematics, statistics and economics are more likely to graduate within the normal time to degree.

The remainder of this paper is organized as follows: the second section provides an overview of the related literature. The conceptual model is presented in the third section, while the institutional context is described in the fourth section. The fifth section provides the methodology used in this study. Our results are presented in section six. Finally, section seven contains our conclusion.

## 2 Literature review

The past two decades witnessed a proliferation of studies that investigated the academic performance of students in tertiary institutions both in terms of grades and excess time to degree. However, the literature on tertiary students’ progression toward the completion of their degrees is US-centric, with very few studies conducted in a European context. Very little, if anything, is known about the duration of study in developing countries. The reason behind the scarcity of studies outside the US and European developed countries is the lack of publicly available micro data on student academic achievement and the difficulty of obtaining access to administrative records in academic institutions. Before we delineate empirical evidence on time to degree and its determinants, a brief preview of the theoretical underpinnings that guided research in this realm is warranted.

### 2.1 Theoretical background

Yue and Fu (2017) posit that the majority of empirical studies on time to degree are rooted in four theoretical models: the interactionist theory of student departure (Tinto, 1975), the geometric model of student persistence and achievement (Swail, 2003), the student attrition model (Bean, 1982; Bean and Metzner, 1985) and the investment model of commitment (Rusbult, 1980). The model developed by Tinto (1975) proposes that college persistence is driven by dynamic interactions between individual students and the academic and social systems of their educational institutions. The model suggests that students enter college with initial goals and commitments that are shaped by some pre-enrollment characteristics, including secondary school grades, family background and the gender of the student. According to the model, the level of success in integrating the academic and social systems of the institution will continuously modify the level of the student's initial goals and commitments.^3^ The modified goals and commitments will affect the student's persistence and determination, which ultimately impact their degree completion time.

The student attrition model initially developed by Bean (1980) draws on the original work of Price (1977) that deals with employee turnover. Bean (1980) suggested that the process of student attrition and employee turnover have much in common, as the reasons behind employee turnover in work organizations closely resemble those of student attrition in academic institutions. Like employees, students' satisfaction, and subsequently their persistence, are affected by organizational factors. In a subsequent study, Bean (1982) developed a model that identified the variables that affect students' intentions to leave, which was posited to be the best predictor of actual dropout in the model. To this end, Bean (1982) categorized the variables that contribute to the student's decision to drop out of college into the following four main categories: background (e.g., age, secondary school GPA), organization (e.g., contacts with faculty, memberships in campus organizations, absenteeism), environment (e.g., finances, family responsibilities) and outcome variables (i.e., attitudinal, such as satisfaction, level of stress and anxiety, and academic outcomes, which are generally measured by GPA).

The Rusbult (1980) investment model of commitment was initially developed to understand the attachment to and continuation of close relationships. Rusbult and Farrell (1983) applied the model to explore job commitment and its link to turnover. Okun et al. (1991), Hatcher et al. (1992) and Okun et al. (2009), among others, utilized the Rusbult (1980) investment model of commitment in the higher education context to predict student persistence and found promising results. According to the investment model, commitment in a relationship is driven by satisfaction, alternatives, and investment. In the context of higher education, students' satisfaction with their college is determined by weighing their received or experienced rewards against their incurred costs of attendance. Alternatives can be other options that the students might consider pursing in lieu of going to college. Among the possible alternatives are attending a different college, starting a small business, or seeking employment. Investment from the student's perspective can be measured by the time they spend attending lectures and studying and by the money they spend in tuition fees and other related expenses.

Swail (2003) posits that the geometric model differs from other models by placing the student at the center of the model. The geometric model postulates that students' persistence is driven by three factors: cognitive, social, and institutional factors. The first set of factors are cognitive, which constitute the student's academic ability, including the level of proficiency in reading, writing, and mathematics. The second set of factors are social factors, including the ability to interact effectively with other persons, personal attitudes, and cultural history. Finally, institutional factors capture the practices, strategies, and culture of colleges or universities that are linked to student persistence and achievement. These factors include faculty teaching quality, academic support and student advising, financial aid, student services, admissions, and curriculum management.

### 2.2 Empirical evidence

Early empirical work on time to completion was concerned largely with postgraduate degrees, particularly doctoral degrees (for example, Booth and Satchell, 1995; Ehrenberg et al., 2007; Siegfried and Stock, 2001; Stock and Siegfried, 2006). The importance of baccalaureate degree completion and time to degree, nonetheless, has been underscored by the increase in the literature on this subject in recent years. This attention is not limited only to academic circles but also extends to policy-makers, as governments are inclined to tie university funding to graduation rates (for a detailed discussion, see Stohs and Schutte, 2019).

As mentioned above, barriers to data accessibility restricted the focus of these studies to only developed countries, particularly the US and European countries, albeit to a lesser extent. Indeed, many empirical studies on degree completion are based on US data drawn from longitudinal studies published by the National Center for Education Statistics (NCES) (Adelman, 1999, 2006; Bound et al., 2012; Lee, 2010; Sublett, 2019; Witteveen and Attewell, 2021). Likewise, studies conducted in an Italian context utilize AlmaLaurea annual survey reports published by AlmaLaurea, the Interuniversity Consortium in Italy and those carried out in Germany employ the Absolventenpanel (panel survey of graduates) 2001 of the HIS (Hochschul-Informations-System) (see, Theune, 2015) and the German Socio-Economic Panel (SOEP) (see, Glocker, 2011), while Häkkinen and Uusitalo (2003) and Häkkinen (2006) use the employment statistics (ES) of Statistics Finland in their Finnish-based studies. However, few studies that use administrative data from a single academic institution in the US (DesJardins et al., 2002, 2003; Lee, 2010; Yue and Fu, 2017), Spain (Lassibille and Navarro Gómez, 2011), and Italy (see, Garibaldi et al., 2012).

While prior studies have aided our understanding of the factors that affect degree completion and time-to-degree, the findings that emerge from these studies are not uniform. The heterogeneity of the findings is not surprising given the differences among studies in the definition of time to degree, the underlying theoretical framework, the sample, and the statistical technique. The extant studies have different focuses given their research context and research question(s).

Early empirical evidence, including DesJardins et al. (2002) and DesJardins et al. (2003), investigates the factors related to student departure and time to degree based on single institution-level data from two US public universities. Using data from the University of Minnesota-Twin Cities campus, DesJardins et al. (2002) focus on the temporal dimension of student departure and the importance of financial aid for reducing the likelihood of dropping out during the first year at college. In a subsequent study, based on data provided by the University of Iowa, DesJardins et al. (2003) show that college academic performance, pre-enrollment academic achievement, and college major were the most significant variables in explaining success. In a salient study, Bound et al. (2012) analyzed the reasons behind the increase in the time to graduate for bachelor's degrees in the US between 1972 and 1988 based on data obtained from longitudinal studies published by the NCES. Their results reveal that two reasons explain the documented increases in time to degree: first, declines in collegiate resources (mainly the decreases in student-faculty ratios that lead to reduction in course offerings needed for degree progress) at non-selective public universities. Second, increases in student employment needed to fund their education preclude their ability to take a full load of classes.

Similar studies that aim to explore the general determinants of time to degree outside the US include Brunello and Winter-Ebmer (2003), Lassibille and Navarro Gómez (2011) and Aina et al. (2011). Using data from the University of Ma'laga and the Ministry of Education of the Autonomous Government of Andalusı'a in Spain, Lassibille and Navarro Gómez (2011) find that less than 40% of higher education degree recipients graduate within the minimum period of time. The results from their analysis indicate that students’ abilities have the strongest impact on time to graduation, followed to a lesser extent by socioeconomic background, motivation when entering the program and gender. The results also show that recipients who received financial support throughout their studies completed their degrees faster; likewise, promoting the academic performance of students at the beginning of the program significantly accelerated student progress toward graduation.

Aina et al. (2011) used data from the AlmaLaurea annual surveys collected from students enrolled in 33 Italian universities. Employing a two-stage estimation procedure, their findings show, based on the first-stage estimation of the probability of graduating at the individual level, that pre-enrollment conditions show the expected signs and are statistically significant (e.g., individuals who have better secondary school grades and more highly educated parents are more likely to complete their degree in a timely manner), while students who rent an accommodation and work to support their studies are less likely to finish their degree on time. The results from the second stage indicate that the probability of timely graduation is positively associated with the human and physical resources available to every university after controlling for all other effects in the first stage.

Using data from a survey conducted in ten European countries covering more than 3,000 students enrolled in 26 economics and business colleges, Brunello and Winter-Ebmer (2003) examined the expected college completion time of college students. The authors’ focus was the role of labor market variables (i.e., unemployment, wage differentials and employment protection and to the funding of tertiary education) in accounting for the observed cross-country variations of the expected college completion time. The results show that the increase in expected college completion time is associated with higher unemployment rates, a narrow college wage gap, a higher share of public spending on higher education, stricter employment protection and the perceived quality of the college.

Other studies have a more specific focus motivated largely by policy initiatives. Häkkinen and Uusitalo (2003) and Glocker (2011) focus on the impact of student aid, while Garibaldi et al. (2012) examine the impact of changes in tuition fees on duration of study. Häkkinen and Uusitalo (2003) utilize employment statistics obtained from Statistics Finland. Their results show that student aid reform in Finland (which aimed to encourage students to complete their degrees on time by distributing more financial aid at a faster rate) had only a modest effect on graduation times. The authors argued that the documented reduction in time to degree is attributed to the sharp increase in the unemployment rate that, in turn, reduced student employment opportunities. Using data from the German Socio-Economic Panel (SOEP), Glocker (2011) shows that while an increase in student aid has no significant effect on time-to-degree, it reduces the likelihood of dropping out. However, student aid recipients (who come from low-income households) finish more quickly than comparable students who are supported by the same amount of parental/private transfers, as financial aid terms and conditions constitute an incentive for graduating faster. Garibaldi et al. (2012), based on single institutional-level data from Bocconi University, use a regression discontinuity design to show that an increase in continuation tuition fees by 1,000 euros is associated with a decline in the probability of late graduation by 5.2% given that the benchmark probability is 80%.

Theune (2015) focuses on the relationship between students’ part-time work and their time to degree in Germany. The author uses data obtained from Absolventenpanel (panel survey of graduates) and finds that part-time work has an increasing effect on time to degree. In a similar vein, Häkkinen (2006) examines the impact of students' employment decisions on their employment and postcollege earnings. The results show that although work experience increases earnings considerably during the first year following graduation, the effect diminishes, losing statistical significance over the later years. The author concludes that, considering that working usually leads to longer times-to-degree, the results show no significant returns to student employment. Aina and Casalone (2020) and Witteveen and Attewell (2021) examine the relationship between delayed time-to-degree and later employment and postcollege earnings. Aina and Casalone (2020), using data from the AlmaLaurea surveys, find that delayed graduation reduces the probability of employment by 0.8% points for each year of delay, and this effect persists even five years after graduation. Noticeably, women and graduates in nonscientific fields are the most severely affected groups. In contrast, the findings based on US data paint a different story. Using data obtained from longitudinal studies published by the NCES entitled Baccalaureate and Beyond (B&B) studies over the period 1993 to 2003 in the US, Witteveen and Attewell (2021) find that while delayed time to degree is not related to employment chances, it correlates with lower postcollege earnings ranging between 8% and 15%, depending on the length of delay. More interestingly, the results show that delayed graduation in combination with working full-time during college has no negative bearing on postcollege earnings.

Yue and Fu (2017) and Huntington-Klein and Gill (2020) examine the influence of students' decisions regarding majors and course load on their performance and graduation time using data from a single university in the US. Based on data collected from a large state university in California, Yue and Fu (2017) find that academic performance has the strongest impact on graduation and time to degree, followed by students' decisions on majors (i.e., enrolling in double majors/minors). In fact, precollege characteristics explain only a very small proportion of the total variation in time to degree after controlling for students' performance and decisions. An interesting finding is that enrollment and enrollment intensity have an overall negative effect on graduation time. Huntington-Klein and Gill (2020) shed light on this finding using data from one of the largest four-year public universities in California. Their goal was to investigate the causal effect of course load on students' grades and GPA. The authors find no evidence that high course loads have a negative impact on student grades (no negative impact on students’ grades when they take 15 credits as opposed to 12 credits in a semester).

Sublett (2019) explores the relationship between enrollment in distance education (DE) courses and time to degree or transfer completion in the context of community colleges in the US. The dataset is collected from the Beginning Postsecondary Students Longitudinal Study 2004–2009 conducted by the NCES. The results show that students who completed DE coursework needed two to three fewer months to complete their bachelor's degrees. Fischer et al. (2021) examine the link between taking online course with degree completion and time-to-degree in the context of large public research university in California. The findings of the study are consistent with those of Sublett (2019) in that taking major-required courses online increases the likelihood of successful 4-year graduation and slightly faster time-to-degree.

Brugiavini et al. (2020) focused on the link between excess time to degree and GPA in the Italian context. Their dataset is constructed by matching the AlmaLaurea survey data with university-level administrative data. The main finding that emerges from this study is that excess time to degree strongly and negatively impacts GPA, possibly because excessively long enrollment in university results in the stock of knowledge becoming obsolete with the passage of time. In a recent study using data from one public, 4-year university in the southeastern U.S., Demeter et al. (2022) develop a machine-learning model to predict whether or not first-time-in-college undergraduates will graduate and when they will graduate based on a plethora of predictors. These predictors include admissions, academic, and financial aid records two to six semesters after enrollment.

## 3 The conceptual model

**Figure 1:**
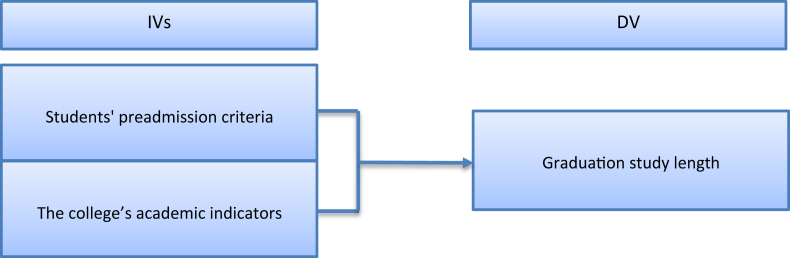
The Study Conceptual Model. Source: By authors.

Based on the preceding section, one can clearly see that the findings from prior studies fall into four broad explanatory dimensions: precollege characteristics, college academic performance, college experiences, and environmental factors. However, several studies mainly considered two broad explanatory dimensions, namely, precollege characteristics and college academic performance (Aina et al., 2011; Lassibille and Navarro Gómez, 2011; Yue and Fu, 2017). We are inclined to take this position for two reasons. First, the purpose of this study was to formulate a predictive model to examine the relationship between students’ preadmission criteria, college academic indicators, and students' actual likelihood of graduating within the normal time to graduation (i.e., 4.5 years). Second, data about the labor market, student mobility, family income and education are not available. As such, the conceptual framework of the current study investigates the relationship between predictor variables and graduation study length, as illustrated in Figure 1.

## 4 Institutional context

The Saudi tertiary education system is largely based on two types of institutions: universities and technical colleges. Universities are largely comprehensive and research-oriented, while technical colleges are vocationally oriented and further linked to the job market. Based on the Saudi qualifications framework, the qualifications offered include associate degrees, bachelor's degrees, master's degrees and doctoral degrees. Additionally, diploma and postgraduate diploma programs (higher diplomas) are offered. Saudi Arabia has 29 public universities and 14 private universities that cover the 13 administrative regions in the Kingdom.

Qassim University (QU) is one of Saudi Arabia's comprehensive public universities. QU was founded as part of the national plan to expand higher education and universities in the Kingdom of Saudi Arabia. Supreme Decree No. 7/B/22042 was issued to establish QU in the 2003–2004 academic year by merging the branches of King Saud University and Imam Muhammad bin Saud Islamic University in Qassim. As of today, QU encompasses 38 colleges that offer 59 bachelor's degree programs, 62 master's programs, and 19 doctoral programs. Areas of study include Sharia, Arabic language, business, social sciences, and scientific, engineering, and health specializations. Since its establishment, the university has experienced remarkable growth in enrollment and significant expansion of faculty and administrative staff. The number of male and female students registered at the university as of the end of 2019 approached 70,513, and there were also 1,794 international students from 80 countries. The number of faculty members reached 3,956, while professional staff numbered 3,354.

Royal approval to establish the College of Business and Economics (CBE) was issued in 1981 under the branch of King Saud University in the Qassim Region. CBE received its inaugural cohort of students at the beginning of the 1982 academic year, and this class successfully graduated in 1986. The female section of CBE was established in 1986, and the first cohort was admitted in 1987. This branch later moved under Qassim University as one of its first established colleges. The CBE has four academic departments that offer various programs: the Department of Management, Information Systems, and Production Management; the Department of Accounting; the Department of Business Administration; and the Department of Economics and Finance. Programs in these four departments are offered at three levels: Bachelor (BBA in MIS&PM, BBA in Accounting, BBA in Business Administration, and BBA in Finance); Master (MBA, M.Sc. in Finance, and M.Sc. in Accounting); and Ph.D. (Business Administration). Currently, 1,643 male students and 1,519 female students are enrolled in CBE undergraduate programs. For postgraduate programs, 36 male students and 94 female students were enrolled.

## 5 Methodology

### 5.1 Data, variables and descriptive statistics

The empirical analysis is based on secondary data collected from students' official academic records stored in the information system of Qassim University. To ensure that the necessary ethical oversight was obtained for this study, the use of the present dataset was approved by the Research Ethics Committee (REC) at Qassim University, approval number 62579/1441. The sample included all finance and accounting graduates of the 2018–2019 cohort. This cohort was specifically selected because it is the first cohort to graduate within the normal time to graduation after the college obtained the AACSB accreditation in 2015, which offered the possibility of tracking the grades of the graduated students in the courses considered in this study over a period that could exceed 4.5 years. The cohort consists of 184 graduated students (excluding 4 students with missing data due to transfers from other universities). No statistical power calculation was conducted prior to the data collection, and the sample size was dictated by data availability. Out of 184 students, the data pertaining to 180 (98%) were used in the empirical analysis, and the researchers of this study accepted this sample size based on their previous experience with this design. The composition of the sample is as follows: 102 female students (56.7% of the total sample) and 78 male students (43.3% of the total sample); the sample included 91 finance graduates (50.6% of the total sample) and 89 accounting graduates (49.4% of the total sample).

In light of the theoretical considerations and empirical evidence in the ‘‘Literature review’’ section and the justifications provided in the preceding section, the variables included in the analysis are delineated in the sequel. The dependent variable (i.e., study length) is defined categorically based on student graduation falling within one of three mutually exclusive and exhaustive groups (more, equal, or less than the normal time to degree). We employ a trichotomous, rather than the widely used dichotomous, classification (i.e., within and more than normal time to degree) because the students who complete their degree earlier than expected constitute an important case that warrants further investigation. On the other hand, independent variables are divided into two dimensions: students' preadmission criteria and colleges' academic indicators. The students' preadmission criteria included the students' demographics (i.e., gender and college-entry age) in addition to the preadmission quantitative criteria (i.e., secondary school and scores from the GAT^4^). The college's academic indicators, on the other hand, include the first semester GPA, major, and student achievement in 8 “gatekeeper” courses. These courses are Mathematics in Social Sciences I, Mathematics in Social Sciences II, Business Statistics I, Business Statistics II, Intermediate Accounting I, Financial Reporting Analysis, Corporate Finance, and Project Feasibility Analysis. Table 1 defines all variables included in our empirical analysis.

**Table 1:** Variables included in the logistic regression model.

Independent variables/predictors	Dependent/Outcome variable
**Preadmission criteria**	**Study Length**
Gender (0 = Female, 1 = Male)	G1 = more than 4.5 years
Percent Secondary School (value of continuance marks out of 100)	G2 = equal to 4.5 years
Percent_GAT (value of continuance marks out of 100)	G3 = less than 4.5 years
College_Entry Age (by years)	
**College criteria**	
First_Semester GPA (value of continuance points out of 5)	
Major_BBA (0 = Finance, 1 = Accounting)	
**Before specialization courses**	
Math_1 (value of continuance marks out of 100)	
Math_2 (value of continuance marks out of 100)	
Stat_1 (value of continuance marks out of 100)	
Stat_2 (value of continuance marks out of 100)	
**After specialization courses**	
Acct_1 (value of continuance marks out of 100)	
Acct_2 (value of continuance marks out of 100)	
Fin (value of continuance marks out of 100)	
Econ (value of continuance marks out of 100)	

**Source**: By authors.

### 5.2 The statistical approach

Several studies, including DesJardins et al. (2003), Adelman (2006) and Brugiavini et al. (2020), use discrete choice regression models to analyze the determinants of the duration of study. Because ordinary least squares (OLS) is not the proper statistical method for binary variables, logistic or probit estimation techniques are employed. Since we adopt a trichotomous classification for the dependent variable (graduation time), the multinomial logistic regression (MLR) model, discussed for example in Hosmer et al. (2013), was applied to investigate the extent of predictability of the graduation study length for students. Y is the dependent variable G, with K=3 categories. Then, the multinomial regression consists of a system of 3 logistic models that, after being standardized for the reference category of Y (here, the reference category is "G1 = more than 4.5 years" because it was the largest category), enable us to compute the probability of Y taking the value of each of the 3 categories. Following Hosmer et al. (2013, p. 270-271), We use matrix notation, where Χ is the matrix of the independent variables and β is the vector of the coefficients, the probabilities of Y are given by

(1) $$P\left(Y=1|\text{Χ}\right)=\frac{1}{1+\sum_{k=2}^{3}e^{\text{Χ}{\text{β}}_{k}}},\;P\left(Y=2|\text{Χ}\right)=\frac{e^{\text{Χ}{\text{β}}_{2}}}{1+\sum_{k=2}^{3}e^{\text{Χ}{\text{β}}_{k}}},\;P\left(Y=3|\text{Χ}\right)=\frac{e^{\text{Χ}{\text{β}}_{3}}}{1+\sum_{k=2}^{3}e^{\text{Χ}{\text{β}}_{k}}}$$

The K−1=2 odds of each category of Y, relative to the reference category, are given by

(2) $$\frac{P\left(Y=2|\text{Χ}\right)}{P\left(Y=1|\text{Χ}\right)}=e^{\text{Χ}{\text{β}}_{2}},\;\frac{P\left(Y=3|\text{Χ}\right)}{P\left(Y=1|\text{Χ}\right)}=e^{\text{Χ}{\text{β}}_{3}}$$

and by taking the natural logarithm for the two members of the equality, we obtain 2 log odds, relative to the reference category that represents the multiequation model,

(3) $$\ln\frac{P\left(Y=2|\text{Χ}\right)}{P\left(Y=1|\text{Χ}\right)}=\text{Χ}{\text{β}}_{2},\text{ln}\frac{P\left(Y=3|\text{Χ}\right)}{P\left(Y=1|\text{Χ}\right)}=\text{Χ}{\text{β}}_{3}$$

The logit for each nonreference category against the reference category depends on a set of explanatory variables. The model is estimated using the maximum likelihood method.

## 6 Results

The dataset was analyzed using IBM SPSS version 22 (IBM, 2013). Table 2 presents the descriptive statistics of the sampled data as well as the distribution of finance and accounting graduates according to grades in quantitative courses (before specialization) and specialized courses (those taught jointly to finance and accounting students according to the curriculum of the CBE). The data are presented as the means, standard deviation (SD), and frequency counts (%).

**Table 2:** Summary descriptive statistics for the sampled data.

Variable	Total sample (N = 180)
**Independent variables**	
Preadmission criteria	
Mean Percent_Secondary School (SD)	92.94 (5.99)
Mean Percent_GAT (SD)	74.44 (7.89)
Mean College_Entry Age (SD)	17.73 (1.15)
Gender	
Female, n. (%)	102 (56.7)
Male, n. (%)	78 (43.3)
**College criteria**	
Mean First_Semester GPA (SD)	3.71 (0.86)
Major_BBA	
Finance, n. (%)	91 (50.6)
Accounting, n. (%)	89 (49.4)
Mean Math_1 (SD)	77.77 (11.18)
Mean Math_2 (SD)	76.76 (12.96)
Mean Stat_1 (SD)	79.64 (12.44)
Mean Stat_2 (SD)	86.09 (11.32)
Mean Acct_1 (SD)	70.80 (10.37)
Mean Acct_2 (SD)	77.46 (9.49)
Mean Fin (SD)	79 (12)
Mean Econ (SD)	81 (9.07)
**Dependent variable**	
Study length	
G1 > 4.5 Y, n. (%)	88 (48.9)
G2 = 4.5 Y, n. (%)	59 (32.8)
G3 < 4.5 Y, n. (%)	33 (18.3)

**Source:** data processed by authors (2020); **Note:** GAT, General Aptitude Test; GPA, grade point average; BBA, Bachelor of Business Administration; preadmission data (students' performance in secondary school, student performance in GAT, college entry age, and gender), college data (the first semester GPA, specialization in BBA, Math_1, Mathematics in Social Sciences I; Math_2, Mathematics in Social Sciences II; Stat_1, Business Statistics I; Stat_2, Business Statistics II; Acct_1, Intermediate Accounting I, Acct_2, Financial Reporting Analysis; Fin, Corporate Finance; Econ, Project Feasibility Analysis course; SD, Standard Deviation.

Table 2 reveals that slightly more than half of students graduate within normal time to degree (32.8% + 18.3% = 51.1%). This implies that there is great room for improvement. This motivates our attempt to formulate a predictive model to examine the relationship between students’ preadmission criteria, college academic indicators, and students' actual likelihood of graduating within the normal time to graduation (i.e., 4.5 years). The results of the likelihood ratio test for each of the independent variables are presented in Table 3.

**Table 3:** The likelihood ratio tests for independent variables.

Independent variables	$\chi^{2}$	df	p value
Percent_Secondary School	10.601	2	.005∗∗
Percent_GAT	6.779	2	.034∗
College_Entry Age	9.909	2	.007∗∗
First_Semester GPA	2.975	2	.226
Math_1	3.280	2	.194
Math_2	6.070	2	.048∗
Stat_1	10.026	2	.007∗∗
Stat_2	2.470	2	.291
Acct_1	3.320	2	.190
Acct_2	2.779	2	.249
Fin	.407	2	.816
Econ	19.498	2	.000∗∗
Gender	3.463	2	.177
Major_BBA	.219	2	.896

**Source:** data processed by authors (2020); ∗p < 0.05, ∗∗p < 0.01.

Based on Table 3, we can determine the independent variables that have a significant impact on study length. Out of the 14 independent variables considered, only 6 were found to be statistically significant. These statistically significant variables are Percent_Secondary School, Percent_GAT, College_Entry Age, Math_2, Stat_1, and Econ. To construct a parsimonious model, we exclude the insignificant variables in our sample.

Parameter estimates for both models: the full model (with all the independent variables) and the reduced model (with only the significant independent variables) are presented in Table 4. The model fit statistics at the bottom of Table 4 include the likelihood ratio test and -2 log likelihood statistic for both the full and the reduced model. The likelihood ratio test is computed for both models against the null model (with only a constant, while all the parameter coefficients are restricted to zero). The likelihood ratio test is statistically significant at the 1% level for both models. Thus, the independent variables, as a group, contribute significantly to the prediction of the study length. Furthermore, three different pseudo $R^{2}$ statistics (i.e., Cox and Snell, Nagelkerke and McFadden) are reported to assess model fit by determining the effect size of the model. Like $R^{2}$, higher values of pseudo $R^{2}$ indicate a better overall fit of the model to the observations. Values from .2 to .4 for McFadden are considered “highly satisfactory” (see Petrucci (2009, p. 200) and the references therein). Therefore, both models have a satisfactory goodness-of-fit.

**Table 4:** Parameter estimates.

Independent variables/Predictors	The full model		The reduced model	
	G2 vs. G1	G3 vs. G1	G2 vs. G1	G3 vs. G1
	Beta (Wald) (Odds ratio) *p*	Beta (Wald) (Odds ratio) *p*	Beta (Wald) (Odds ratio) *p*	Beta (Wald) (Odds ratio) *p*
**Percent_Secondary School**	.077 (2.337) (1.08) .126	.292 (6.301) (1.34) .012∗	.086 (3.611) (1.09) .057	.252 (9.092) (1.29) .003∗∗
**Percent_GAT**	.046 (1.625) (1.05) .202	.141 (6.223) (1.15) .013∗	.044 (1.767) (1.05) .184	.171 (13.665) (1.19) .000∗∗∗
**College_Entry Age**	.270 (1.571) (1.31) .210	.990 (10.253) (2.69) .001∗∗	.181 (.808) (1.20) .369	.688 (8.325) (1.99) .004∗∗
First_Semester GPA	-.110 (.054) (0.90) .816	1.470 (2.434) (4.35) .119		
Math_1	.046 (2.178) (1.05) .140	.075 (2.293) (1.08) .130		
**Math_2**	-.063 (4.602) (.94) .032∗	.005 (.010) (1.01) .919	-.028 (1.480) (.97) .224	.077 (4.791) (1.08) .029∗
**Stat_1**	.071 (6.503) (1.07) .011∗	-.050 (.936) (.95) .333	.084 (12.357) (1.09) .000∗∗∗	.039 (1.170) (1.04) .279
Stat_2	.046 (1.973) (1.05) .160	.060 (.886) (1.06) .347		
Acct_1	-.014 (.181) (.99) .670	.067 (1.982) (1.07) .159		
Acct_2	.066 (2.534) (1.07) .111	.054 (.889) (1.06) .346		
Fin	.009 (.055) (1.01) .814	.034 (.403) (1.04) .525		
**Econ**	.094 (6.600) (1.10) .010∗	-.107 (4.152) (.90) .042∗	.123 (16.074) (1.13) .000∗∗∗	-.031 (.678) (.97) .410
Gender (0 = Female, 1 = Male)	.671 (1.692) (1.96) .193	-.581 (.572) (.56) .450		
Major_BBA (0 = Finance, 1 = Accounting)	.202 (.147) (1.22) .702	-.043 (.003) (.96) .953		
**Model Fit**				
Likelihood ratio test	$\chi^{2}=179.477,\;p=.000^{***}$		$\chi^{2}=143.188,\;p=.000^{***}$	
-2 log likelihood	190.058		226.346	
**Goodness of Fit**				
Pseudo $R^{2}$	Cox and Snell = .631, Nagelkerke = .724, McFadden = .486		Cox and Snell = .549, Nagelkerke = .629, McFadden = .387	

**Source:** data processed by authors (2020); ∗p < 0.05, ∗∗p < 0.01, ∗∗∗p < 0.001. Note: The authors round the odds ratio to two significant digits if the leading nonzero digit is four or more; otherwise, they round to three. “For more information about the Rule of Four, see Cole (2015)”.

Table 4 contains the parameter estimates for the full and final reduced models. The reference category is G1 (graduating in more than 4.5 years), against which the other two categories, G2 (graduating exactly in 4.5 years) and G3 (graduating in less than 4.5 years), are compared. Both the full and the reduced models have two sets of estimated parameters: the first pertains to the G2 category relative to the reference category, and the other pertains to the G3 category relative to the reference category.

While we report the results for both models (the full and the reduced), we base our interpretation on the reduced model. The parameters estimated for the reduced model are shown in the last two columns of Table 4. The comparison between G2 vs. G1, that is, graduating in exactly 4.5 years and delayed graduation, indicates that only two out of the six variables considered are statistically significant. The significant variables are Stat_1 and Econ, while none of the students' preadmission criteria displayed any statistically meaningful effect except for Percent_Secondary School, which is marginally significant at the 10% level. A word on the interpretation of the significant parameters could be useful. The results illustrate that when controlling for the other variables, a one-mark incremental increase in the student's grades in Stat_1 and Econ increased the odds of graduating on time, rather than experiencing delayed graduation, by 9% and 13%, respectively.

Turning to the last column in Table 4 regarding the comparison between G3 vs. G1, that is, graduating in less than 4.5 years and delayed graduation, we see that 4 out of 6 variables are statistically significant. Three out of the 4 significant variables fall in the students' preadmission criteria dimension, including the following: Percent_Secondary School, Percent_GAT and College_Entry Age. However, Math_2 was the only college academic indicator to display statistical significance. These results are interpreted as follows: when other variables are held constant, a one-percentage-point incremental increase in the student's performance in secondary school and the GAT test increased the odds of graduating earlier than 4.5 years, rather than experiencing delayed graduation, by 29% and 19%, respectively. Notably, a one-year increase in the student's age upon entry to college doubled the odds of graduating early. On the other hand, a one-mark incremental increase in the student's grades in Math_2 increased the odds of graduating early.

**Figure 2:**
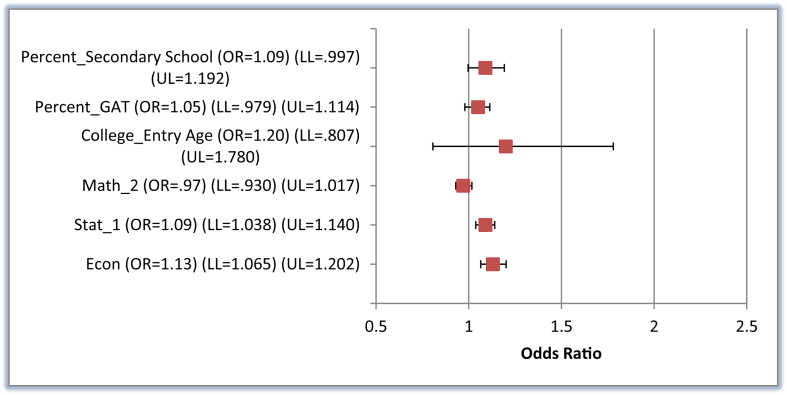
Odds Ratios for G2 vs. G1 Reduced Model Predictors with 95% Wald CI. Source: data processed by authors (2020); Note: G2 = Graduation study length equal to 4.5 years, G1 = Graduation study length more than 4.5 years, CI = Confidence Interval, OR = Odds Ratio, LL = Lower Limit, and UL = Upper Limit.

**Figure 3:**
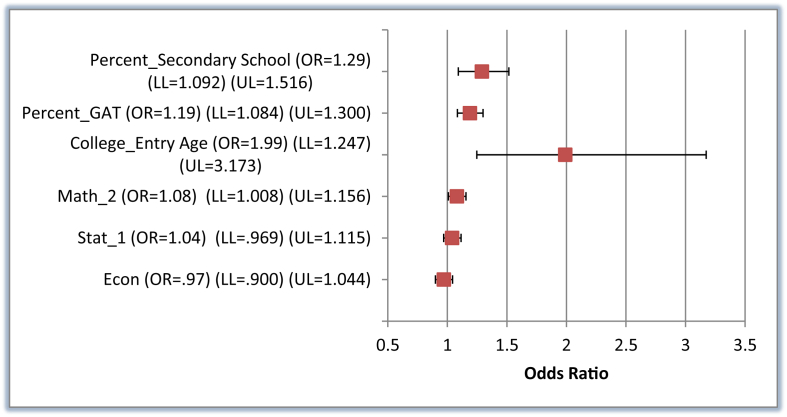
Odds Ratios for G3 vs. G1 Reduced Model Predictors with 95% Wald CI. Source: data processed by authors (2020); Note: G3 = Graduation study length less than 4.5 years, G1 = Graduation study length more than 4.5 years, CI = Confidence Interval, OR = Odds Ratio, LL = Lower Limit, and UL = Upper Limit.

Figure 2 illustrates the odds ratios for G2 vs. G1 model predictors, and Figure 3 shows the odds ratios for the G3 vs. G1 model predictors.

The classification table indicates the usefulness of the estimated model by comparing the observed (actual frequencies in the data) versus the predicted groupings obtained by the means of the estimated model. The classification table results are presented in Table 5. Looking at Table 5, one can see that the diagonals (in bold) report the correctly classified cases for both the full and reduced models. Overall, we obtained 81.1% prediction precision for the full model and 75.6% for the reduced model. Precise predictions were more frequent for the G1 category (at 89.8% for the full model and 83% for the reduced model) than they were for the G2 category (at 72.9% for the full model and 69.5% for the reduced model) and the G3 category (at 72.7% for the full model and 66.7% for the reduced model). The fact that G1, which is the largest group, produces the best prediction is common when using MLR (Hosmer et al., 2013).

**Table 5:** The power of classification.

Observed (marginal %)	Predicted							
	The full model				The reduced Model			
	G1	G2	G3	Percent Correct	G1	G2	G3	Percent Correct
G1 (48.9%)	**79**	6	3	89.8%	**73**	12	3	83.0%
G2 (32.8%)	13	**43**	3	72.9%	14	**41**	4	69.5%
G3 (18.3%)	5	4	**24**	72.7%	6	5	**22**	66.7%
Overall Percentage	53.9%	29.4%	16.7%	81.1%	51.7%	32.2%	16.1%	75.6%

**Source:** data processed by authors (2020).

The usefulness of the full and reduced models is evaluated by computing the proportional by chance accuracy rate of the classification (for example see Petrucci (2009)). As an acceptable rule of thumb, an overall classification accuracy rate that is greater than the proportional by chance accuracy rate by 25% or more implies that the model has adequate accuracy.

The results in Table 5 show that the criterion for model classification accuracy is satisfied, and that the model is adequate. Because the overall classification accuracy rate ranges from 81.1% to 75.6% for the full and reduced models, respectively, the accuracy rate is greater than the proportional by chance accuracy criteria by 43.1% for the full model and by 37.6% for the reduced model.

## 7 Conclusion

This study aims to explore the predictors of the study length of a bachelor's degree in business administration (BBA). The data for our analysis come from administrative records for all finance and accounting graduates of the 2018–2019 cohort at the College of Business and Economics in Qassim University. We employ the MLR to explore the predictors of the study length. The descriptive statistics show that slightly over half (51.1%) of the graduates complete their degree within the normal time to degree.

The results obtained from the MLR indicate that both the student's preadmission criteria and their college academic indicators have predictive power for study length, as we found that higher marks in “gatekeeper” quantitative courses, including Business Statistics I and Project Feasibility Analysis, were associated with increased odds of graduation within the normal time to degree (4.5 years). In addition, we found that higher Percent in Secondary School, higher Percent GAT score, higher College Entry Age, and higher marks in Mathematics in Social Sciences II were associated with increased odds of an earlier graduation than the normal time to degree. Overall, our results are not surprising, as accounting and finance courses, by their very nature, are quantitative and analytical, which explains the predictive power that quantitative courses possess for explaining study length.

Several implications for the development of education policy can be derived from the above findings. The most important is the need to support academic progression toward degree completion within the normal time to degree. This is achieved by periodically reviewing policies and procedures for student admissions, curriculum design and assurance of learning and academic advising considering empirical data analysis on student progress. Such data-driven policies and procedures offer early identification of retention and progression issues that contribute to a more well-informed decision-making process. The corrective course of action in response to retention and progression issues ranges from short-run intervention with support and counseling (or students' dismissal from programs if necessary) to long-run changes, including revising curriculum and admission criteria. The goal of these policies and procedures is to reduce academic failure and its negative consequences on students’ emotional and financial wellbeing and to the associated resource and performance implications for higher education institutions and the greater society.

This study has several caveats. First, only finance and accounting graduates were included in the sample. Second, the dataset included only one cohort of graduates (2018/2019). Third, several dimensions that may be related to the graduation study length are not included in the empirical analysis, such as environmental factors (finances, family responsibilities, employment status) and socioeconomic status. Future research using a larger sample size of students is needed to extend this type of research (including all majors in the same business schools), apply the model in other business schools in Saudi Arabia, and include other colleges with the same business educational curricula outside Saudi Arabia to determine whether the findings of this research hold.

## Declarations

### Author contribution statement

Nassar S. Al-Nassar: Conceived and designed the experiments; Performed the experiments; Analyzed and interpreted the data; Contributed reagents, materials, analysis tools or data; Wrote the paper.

Ahmed A. Alhajjaj: Conceived and designed the experiments; Performed the experiments; Analyzed and interpreted the data; Contributed reagents, materials, analysis tools or data.

Abbas Bleady: Conceived and designed the experiments; Performed the experiments; Analyzed and interpreted the data; Contributed reagents, materials, analysis tools or data.

### Funding statement

This research did not receive any specific grant from funding agencies in the public, commercial, or not-for-profit sectors.

### Data availability statement

The data that has been used is confidential.

### Declaration of interest's statement

The authors declare no conflict of interest.

### Additional information

No additional information is available for this paper.

## Acknowledgements

We would like to thank the editor and anonymous reviewers for their detailed and insightful comments and suggestions that helped to improve the quality of the paper. All remaining errors are ours alone. The views expressed in this paper are those of the authors and do not necessarily reflect those of Qassim University.

## Footnotes

1 The U.S. News and World Report Best Colleges Rankings, which allows for 35 percent of a school's rank to derive from “its success at retaining and graduating students within 150 percent of normal time (six years)” (U.S. News and World Report 2019).

2 Such data are routinely used to generate standardized KPIs (Zhu, 2004). In Saudi Arabia, KPIs have been increasingly used by the ministry of education as well as national and international accreditation bodies to measure the performance of colleges and schools relative to their peers.

3 Academic integration can be measured by the students' academic performance, i.e., GPA. Social integration can be measured by students' interaction with college community by participating in extracurricular activities and students' clubs and associations in addition to peer friendships on campus and relationships with instructors outside of class.

4 The GAT is a standard mandatory test for high-school students, which aids Saudi universities in selecting applicants who meet their requirements and are more qualified to continue their studies in these universities (ETEC, 2020).
